# A COMPARISON OF MELANCHOLIC AND NONMELANCHOLIC RECURRENT MAJOR DEPRESSION IN HAN CHINESE WOMEN

**DOI:** 10.1002/da.20875

**Published:** 2011-11-07

**Authors:** Ning Sun, Yihan Li, Yiyun Cai, Jing Chen, Yuan Shen, Jing Sun, Zheng Zhang, Jiulong Zhang, Lina Wang, Liyang Guo, Lei Yang, Li Qiang, Yanchun Yang, Gang Wang, Bo Du, Jing Xia, Han Rong, Zhaoyu Gan, Bin Hu, Jiyang Pan, Chang Li, Shufan Sun, Wei Han, Xue Xiao, Lei Dai, Guixing Jin, Yutang Zhang, Lixin Sun, Yunchun Chen, Haiying Zhao, Yamei Dang, Shenxun Shi, Kenneth S Kendler, Jonathan Flint, Kerang Zhang

**Affiliations:** 1 No.1 Hospital of Shanxi Medical UniversityTaiyuan, Shanxi, People's Republic of China; 2 Wellcome Trust Centre for Human GeneticsOxford, United Kingdom; 3 Fudan University affiliated Huashan HospitalShanghai, People's Republic of China; 4 Shanghai Jiao Tong University School of Medicine affiliated Shanghai Mental Health CentreShanghai, People's Republic of China; 5 Shanghai Tongji University affiliated Tongji HospitalShanghai, People's Republic of China; 6 Nanjing Brain HospitalNanjing, Jiang Su, People's Republic of China; 7 No. 4 Affiliated Hospital of Jiangsu UniversityZhenjiang, Jiang Su, People's Republic of China; 8 Tianjin Anding HospitalHexi District, Tianjin, People's Republic of China; 9 Shandong Mental Health CenterJinan, Shan Dong, People's Republic of China; 10 No. 1 Hospital of Medical College of Xian Jiaotong UniversityXian, Shan Xi, People's Republic of China; 11 No.1 Hospital of Zhengzhou UniversityZhengzhou, He Nan, People's Republic of China; 12 No. 1 Mental Health Center Affiliated Harbin Medical UniversityNangang District, Haerbin, Heilongjiang, People's Republic of China; 13 Mental Health Center of West China Hospital of Sichuan UniversityWu Hou District, Chengdu, Si Chuan, People's Republic of China; 14 Beijing Anding Hospital, Capital Medical UniversityXicheng District, Beijing, People's Republic of China; 15 Hebei Mental Health CenterBaoding, Hebei, People's Republic of China; 16 ShengJing Hospital of China Medical UniversityHeping District Shenyang, Liaoning, People's Republic of China; 17 Shenzhen Kangning HospitalLuo Hu, Shenzhen, People's Republic of China; 18 No. 3 Affiliated Hospital of Zhongshan UniversityTian He District, Guangzhou, Guangdong, People's Republic of China; 19 Mental Hospital of Jiangxi ProvinceNanchang, Jiangxi, People's Republic of China; 20 The First Affiliated Hospital of Jinan UniversityTian He District, Guangzhou, People's Republic of China; 21 Wuhan Mental Health CenterWuhan, People's Republic of China; 22 No.3 Hospital of Heilongjiang ProvinceBeian, Heilongjiang, People's Republic of China; 23 Jilin Brain HospitalSiping, Jilin, People's Republic of China; 24 The First Hospital of China Medical UniversityHe Ping District, Shenyang, People's Republic of China; 25 Dalian No. 7 People's Hospital & Dalian Mental Health CenterGan Jing Zi District, Dalian, People's Republic of China; 26 The First Hospital of Hebei Medical UniversityShijiazhuang, People's Republic of China; 27 Lanzhou University Second Hospital, Second Clinical Medical College of Lanzhou UniversityLanzhou, Gansu Province, People's Republic of China; 28 Psychiatric Hospital of Henan ProvinceXin Xiang, Henan, People's Republic of China; 29 The Fourth Military Medical University affiliated Xijing HospitalXi'an, Shaanxi, People's Republic of China; 30 No. 4 People's Hospital of LiaochengLiaocheng, Shandong, People's Republic of China; 31 Guangzhou Brain Hospital/Guangzhou Psychiatric HospitalFang Cun Da Dao, Li Wan District, Guangzhou, People's Republic of China; 32 Virginia Commonwealth University, Department of Psychiatry, Virginia Institute for Psychiatric and Behavioral GeneticsRichmond, Virginia

**Keywords:** major depression, melancholia, symptom, stressful life events

## Abstract

**Background:**

Although the diagnosis of melancholia has had a long history, the validity of the current DSM-IV definition remains contentious. We report here the first detailed comparison of melancholic and nonmelancholic major depression (MD) in a Chinese population examining in particular whether these two forms of MD differ quantitatively or qualitatively.

**Methods:**

DSM-IV criteria for melancholia were applied to 1,970 Han Chinese women with recurrent MD recruited from 53 provincial mental health centers and psychiatric departments of general medical hospitals in 41 cities. Statistical analyses, utilizing Student's *t*-tests and Pearson's χ^2^, were calculated using SPSS 13.0.

**Results:**

Melancholic patients with MD were distinguished from nonmelancholic by being older, having a later age at onset, more episodes of illness and meeting more A criteria. They also had higher levels of neuroticism and rates of lifetime generalized anxiety disorder, panic disorder, and social and agoraphobia. They had significantly lower rates of childhood sexual abuse but did not differ on other stressful life events or rates of MD in their families.

**Discussion:**

Consistent with most prior findings in European and US populations, we find that melancholia is a more clinically severe syndrome than nonmelancholic depression with higher rates of comorbidity. The evidence that it is a more “biological” or qualitatively distinct syndrome, however, is mixed.

## INTRODUCTION

Melancholia is one of the oldest and most frequently studied subtypes of major depression (MD).[1–5] Its symptoms according to DSM-IV include anhedonia, nonreactivity, psychomotor disturbance, cognitive slowing, diurnal variation of mood, terminal insomnia, appetite, and weight loss.[6] Some have argued that melancholia is an etiologically distinct form of MD,[7–9] that it should be included in DSM-V as a separate mood disorder[10] and that it possesses a distinctive biological etiology with characteristic laboratory test markers.[3]

Several studies have identified characteristic features that distinguish melancholia from MD, including overall severity, a later age of onset, and higher rates of comorbidity.[5, 11, 12] For example, Kendler et al.[11] found that patients with melancholia had higher rates of comorbidity, a larger number of episodes, lower levels of neuroticism, and an increased risk of MD in co-twins. However, from a familial perspective, the differences between melancholic and nonmelancholic MD was quantitative rather than qualitative. In other words, their results were most consistent with the hypothesis that melancholic MD is more severe than, but not etiologically distinct from, nonmelancholic MD.

All the major studies comparing melancholic and nonmelancholic depression have been carried out in European populations. It is not known to what extent current findings will apply to patients in Asia. The aim of this study is to investigate, for the first time to our knowledge, whether features reported to characterize melancholia in the United States and European studies are to be found in China. These features are differences in the clinical symptoms of MD,[7, 9] comorbidity,[5, 11–13] personality,[11] family history,[11] and environment sensitivity.[14] We set out to test not only whether melancholia has characteristic clinical features but also whether it has the same relationship to two known environmental precipitants of MD: stressful life events (SLEs)[15] and childhood sexual abuse (CSA).[16] We carried out our study in a large clinically acquired cohort of women with recurrent MD, all of whom are from the largest ethnic group in China, the Han Chinese. Our major goal is to clarify in this population the degree to which melancholia is quantitatively more severe than, or qualitatively distinct from, nonmelancholic depression.

## SUBJECTS AND METHODS

### STUDY SUBJECTS

The data for this study were drawn from the ongoing China, Oxford, and VCU Experimental Research on Genetic Epidemiology (CONVERGE) study of MD (MD). These analyses were based on a total of 1,970 cases recruited from 53 provincial mental health centers and psychiatric departments of general medical hospitals in 41 cities in 19 provinces and four central cities: Beijng, Shanghai, Tianjin, and Chongqing. All cases were female and had four Han Chinese grandparents. Cases were excluded if they had a pre-existing history of bipolar disorder, any type of psychosis, or mental retardation. Cases were aged between 30 and 60 years, had two or more episodes of MD, with the first episode occurring between 14 and 50 and had not abused drug or alcohol before the first episode of MD. The mean age (and *SD*) of cases in the data set was 45.1 (8.8).

Cases were interviewed using a computerized assessment system. All interviewers were trained by the CONVERGE team for a minimum of 1 week in the use of the interview. The interview includes assessment of psychopathology, demographic and personal characteristics, and psychosocial functioning. The study protocol was approved centrally by the Ethical Review Board of Oxford University and the ethics committee in participating hospitals in China.

### MEASURES

The diagnoses of depressive (dysthymia and major depressive disorder, melancholic and nonmelancholic depression) and anxiety disorders (generalized anxiety disorder (GAD), panic disorder (PD) with or without agoraphobia) were established with the Composite International Diagnostic Interview, which classifies diagnoses according to the Diagnostic and Statistical Manual of Mental Disorders (DSM-IV) criteria.[6] The interview was originally translated into Mandarin by a team of psychiatrists in Shanghai Mental Health Centre with the translation reviewed and modified by members of the CONVERGE team. Phobias, divided into five subtypes (animal, situational, social, blood-injury, and agoraphobia) were diagnosed using an adaptation of DSM-III criteria[17] requiring one or more unreasonable fears, including fears of different animals, social phobia, and agoraphobia that objectively interfered with the respondent's life. The section on the assessment of phobias was translated by the CONVERGE team from the interview used in the Virginia Adult Twin Study of Psychiatric and Substance Use Disorders (VATSPSUD).[18] All conditions were assessed by asking about lifetime episodes.

SLEs were assessed by asking about 16 traumatic lifetime events and the age at their occurrence. The CSA was a shortened version of the detailed module used in the VATSPSUD study, which was in turn based on the instrument developed by Martin et al.[19] and included three factors that were nongenital, genital, and intercourse.

The case interview was fully computerized into a bilingual system of Mandarin and English developed in house in Oxford, and called SysQ. Skip patterns were built into SysQ. Interviews were administered by trained interviewers and entered offline in real time onto SysQ, which is installed in the laptops. Once an interview is completed, a backup file containing all the previously entered interview data can be generated with database compatible format. The backup file together with an audio recording of the entire interview is uploaded to a designated server currently maintained in Beijing by a service provider. All the uploaded files in the Beijing server are then transferred to an Oxford server quarterly.

### STATISTICAL ANALYSIS

Sociodemographic and clinical characteristics of the sample were analyzed. For continuous variables, independent Student's *t*-tests were performed; for categorical variables, Pearson's χ^2^ were calculated. All the characteristics of individuals with melancholic versus nonmelancholic MD were assessed by logistic regression, with melancholia as the dependent variable (0 = absence and 1 = presence). Associations between variables were expressed as odds ratios (OR) and 95% confidence intervals (95% CI). SPSS 13.0 for Windows was used in data analysis.

## RESULTS

We obtained 1,970 cases of recurrent MD from 53 hospitals in China. 81.3% of our cases (*n* = 1,602) met DSM-IV criteria for melancholia during their worst lifetime episode. In this, and subsequent analyses, we carried out a within-case study to determine if there are any characteristics that differentiate those with melancholic MD from those with nonmelancholic MD.

Clinical characteristics of MD cases with and without melancholia are shown in Table 1. Compared with individuals with nonmelancholic MD, the melancholic group was significantly older, and had a later age of onset. Those with melancholia met a significantly higher number of DSM-IV criteria for MD; during the worst depressive episode, the melancholic group were significantly more likely to report feeling hopeless, crying, helpless, nervous and to have experienced functional impairment than nonmelancholic depressives. In addition, the melancholic group had significantly higher neuroticism scores and a more recurrent course than nonmelancholic patients. However, the two groups did not differ in the length of the longest episodes or in the risk of having a positive family.

**TABLE 1:** Clinical characteristics in female with melancholic versus nonmelancholic patients with MD

Characteristics	Melancholic *n* = 1,602; 81.3%	Nonmelancholic *n* = 368; 18.7%	OR	95% CI	*P*-value
Age	45.5 ± 8.8	43.4 ± 8.7	1.03	1.01–1.01	.003
Age of onset, years	36.2 ± 10.0	34.3 ± 9.5	1.02	1.01–1.03	.003
Number of episode	4.4 ± 5.4	3.5 ± 3.1	1.06	1.02–1.09	.005
Length of longest episode	46.7 ± 91.2	49.5 ± 93.5	1.00	0.99–1.00	.60
No. of DSM-IV A criteria	8.5 ± 0.8	7.2 ± 1.4	2.75	2.43–3.10	<.001
Feeling irritable	1,172 (71.3)	244 (73.5)	0.99	0.76–1.30	.96
Feeling hopeless	1,326 (83.0)	213 (64.2)	2.72	2.10–3.53	<.001
Crying	1,060 (66.4)	202 (60.8)	1.27	0.99–1.62	.06
Feeling helpless	1,437 (89.9)	266 (80.1)	2.22	1.62–3.03	<.001
Feeling nervous	1,451 (90.8)	279 (84.0)	1.88	1.34–2.63	<.001
No known precipitant	1,054 (66.0)	229 (69.0)	0.87	0.67–1.12	.30
Functional impairment	1,386 (87.0)	230 (69.5)	2.93	2.22–3.86	<.001
Neuroticism	12.7 ± 6.1	11.0 ± 5.9	1.05	1.03–1.07	<.001
Family history of MD	513 (33.1)	112 (31.9)	1.06	0.82–1.35	.70

Odds ratios (OR), *P*-values, and 95% confidence intervals (95% CI) are shown for clinical features of MD. ORs greater than one indicate that the clinical feature or symptom is more common in patients with melancholia. ORs less than one indicate that the clinical feature or symptom is less common in melancholic patients. MD, major depression.

Comorbid rates for other psychiatric disorders in the nonmelancholic and melancholic groups are shown in Table 2. A diagnosis of dysthymia was not associated with a melancholic subtype nor with a diagnosis of animal, situational or blood-injury phobia. Cases of MD with melancholia had significantly higher rates of comorbidity with GAD, PD, social phobia, and agoraphobia.

**TABLE 2:** Pattern of comorbidity in female with melancholic versus nonmelancholic MD

Disorder	Melancholic	Nonmelancholic	OR	95% CI	*P*
Dysthymia	286 (17.9)	68 (19.0)	0.93	0.70–1.25	.64
Generalized anxiety disorder	518 (32.7)	63 (17.7)	2.26	1.69–3.03	<.001
Panic disorder	176 (11.1)	19 (5.3)	2.22	1.37–3.63	<.001
Social phobia	520 (33.4)	88 (24.4)	1.50	1.156–1.96	.002
Agoraphobia	437 (28.0)	74 (21.0)	1.46	1.11–1.94	.007
Animal phobia	878 (56.3)	186 (52.7)	1.15	0.92–1.46	.22
Situational phobia	629 (40.4)	128 (36.2)	1.19	0.94–1.52	.14
Blood phobia	634 (40.7)	133 (37.6)	1.14	0.89–1.45	.30

Odds ratios (OR), *P*-values and and 95% confidence intervals (95% CI) are shown for comorbid disorders. ORs greater than one indicate that the clinical feature or symptom is more common in patients with melancholia. ORs less than one indicate that the clinical feature or symptom is less common in melancholic patients. MD, major depression.

We assessed three groups of environmental risk factors for MD in our sample: lifetime SLEs, parenting and CSA. As depicted in Table 3, while we found no differences in the number of reported SLEs or history of parenting, melancholic patients were highly significantly less likely to report a history of CSA.

**TABLE 3:** Environment sensitivity in female with melancholic versus nonmelancholic MD

Environment sensitivity	Melancholic	Nonmelancholic	OR	95% CI	*P*
Stressful life events	1.7 ± 1.7	1.6 ± 1.6	1.04	0.97–1.12	.30
Parental bonding					
Warmth of mother	18.2 ± 8.3	18.3 ± 8.3	0.99	0.96–1.01	.80
Warmth of father	17.0 ± 8.4	17.2 ± 8.2	0.99	0.98–1.01	.77
Authoritarianism of mother	7.7 ± 4.7	7.9 ± 4.5	0.99	0.97–1.02	.53
Authoritarianism of father	7.8 ± 4.8	7.8 ± 4.5	0.99	0.97–1.02	.94
Protectiveness of mother	8.0 ± 4.4	7.8 ± 4.2	1.01	0.98–1.03	.55
Protectiveness of father	7.4 ± 4.3	7.4 ± 4.1	0.99	0.97–1.03	.87
Childhood sexual abuse	134 (8.6)	55 (15.4)	0.52	0.37–0.72	<.001
Nongenital	50 (3.2)	17 (4.8)	0.66	0.38–1.16	.15
Genital	54 (3.5)	22 (6.2)	0.55	0.33–0.91	.02
Intercourse	30 (1.9)	16 (4.5)	0.42	0.23–0.77	.006

Odds ratios (OR), *P*-values and and 95% confidence intervals (95% CI) are shown for life events (including childhood sexual abuse) and for three measures of parenting, taken from the parental bonding instrument (warmth, authoritarianism and protectiveness), in each cases assessed in the mother and father. ORs greater than one indicate that the clinical feature or symptom is more common in patients with melancholia. ORs less than one indicate that the clinical feature or symptom is less common in melancholic patients. MD, major depression.

## DISCUSSION

There has been a resurgence of interest in the validity of melancholia as a defined mood disorder, with participants at a recent conference on melancholia arguing that it is a distinctive syndrome.[20] Taylor and Fink want to divide depressive disorders into two categories (melancholia and nonmelancholia),[3] but the lack of clear qualitative differences (including abnormal dexamethasone suppression test and treatment response) lead Parker to propose a hierarchical model that distinguishes psychotic, melancholic, and nonmelancholic subtypes.[21] Evidence from twin studies supports the existence of melancholia as a quantitatively more severe form of depression, with increased comorbidity, greater number of episodes, lower levels of neuroticism, and an increased genetic loading.[11] Validation of melancholia's independent status in a different ethnic group would lend further support to its diagnostic validity and might help to resolve whether it is quantitatively or qualitatively different from MD.

Our results from a large clinical population of Chinese women with recurrent depression consistently support the view that melancholia is a more severe form of MD. The pattern of symptom severity, episode duration and comorbidity echoes that previously reported.[5, 11, 12, 22] Furthermore, our finding that childhood sexual abuse is more common in nonmelancholic depression is consistent with the idea that melancholia has more biological features and approximates to an endogenous depression.[23] However, our findings are not entirely consistent with this hypothesis. CSA was the only life event showing the expected pattern; other SLEs were not significantly different between the two categories. Furthermore, two further predictions from the hypothesis that melancholia reflects a more “biological” and less “environmental/neurotic” form of MD were not supported in our sample. That is, we did not find the predicted increased familial loading for depression[11] nor did we replicate findings of decreased neuroticism in patients with melancholia.[5, 11]

These discrepancies may be due to the greater severity of the features of MD in our sample, rather than reflecting characteristics of MD in Chinese patients. Our cases differ from those used in other studies of melancholia in a number of ways: they were ascertained through hospital in- and outpatient services, required to be aged between 30 and 60 years, with two or more episodes of MD, the first of which had to occur when the patient was older than 14 and younger than 50. Our sample is therefore enriched for severer forms of depression, which, not surprisingly, resulted in high rates of melancholia: 80% of our clinical sample compared to 50% in a US community sample,[11] 44% in a New Zealand outpatient sample,[12] and 32% in a community sample from Switzerland.[5] The cases in our sample who did not meet criteria for melancholia may not have been comparable to nonmelancholic patients in other studies. If, as our data suggest, melancholia differs quantitatively from other forms of depression, our inability to find enrichment for some biological features may simply reflect the fact that our nonmelancholic group was itself comparatively enriched for more severe depression. However, this would not explain the fact that neuroticism scores were higher in the melancholic group. The latter finding may reflect a feature characteristic of the Asian population we have examined and warrants further examination.

In summary, we here report the first detailed comparison, to our knowledge, of melancholic and nonmelancholic MD in an Asian female population. Our findings indicate that compared to nonmelancholic MD, melancholia is, as expected, a generally more severe clinical syndrome. However, support for the hypothesis that melancholia is more “biological” or a qualitatively distinct syndrome from nonmelancholic depression is more mixed. No difference is seen in the risk for MD in families as might be predicted if melancholia were a more “biological/genetic” syndrome. Such a perspective would also predict that melancholic patients should have fewer environmental risk factors. This is seen for childhood sexual abuse but not for overall stressful life events or disturbed parent–child relationships. Finally, melancholic MD with its “biological” roots is sometimes contrasted with “neurotic” depression that is often seen as based in personality disturbances. Our results are clearly inconsistent with this hypothesis. Contrary to what might be expected from this viewpoint, cases of melancholic MD had *higher* levels of the personality trait of neuroticism than nonmelancholic depressives.

Our study has a number of limitations. First, we used a DSM-IV diagnosis of melancholia, many of whose features overlap with MD. We chose this approach because it remains the most widely used, and, as we pointed out earlier, has been used to identify qualitative, as well as quantitative differences from MD.[11] Second, we relied on a cohort that consists entirely of women with recurrent MD in a treatment setting. We cannot be certain that our conclusions will apply to men, or to those with MD outside of hospital. Third, our assessment is based on a single clinical interview, which, while detailed, is subject to the vagaries of remembrance. Fourth, our assessment of SLEs cannot distinguish the degree to which the observed associations between SLEs and melancholia are a result of SLE exposure increasing risk for melancholia or melancholia increasing the risk for SLEs. Fifth, we assessed all conditions for lifetime occurrence, so that they may not always be coterminous with an episode of MD. Consequently, we cannot be certain that conditions are always comorbid. Finally, we do not have estimates of inter-rater reliability of diagnoses; thus, we caution that there is an unspecified degree of error in estimates of the effect sizes that we observe.

## References

[1] Jackson SW (1986). Melancholia and Depression: From Hippocratic Times to Modern Times.

[2] Kendell RE (1976). The classification of depressions: a review of contemporary confusion. Br J Psychiatry.

[3] Taylor MA, Fink M (2008). Restoring melancholia in the classification of mood disorders. J Affect Disord.

[4] Cole J, McGuffin P, Farmer AE (2008). The classification of depression: are we still confused?. Br J Psychiatry.

[5] Angst J, Gamma A, Benazzi F (2007). Melancholia and atypical depression in the Zurich study: epidemiology, clinical characteristics, course, comorbidity and personality. Acta Psychiatr Scand Suppl.

[6] Association AP (1994). Diagnostic and Statistical Manual of Mental Disorders.

[7] Zimmerman M, Black DW, Coryell W (1989). Diagnostic criteria for melancholia. The comparative validity of DSM-III and DSM-III-R. Arch Gen Psychiatry.

[8] Zimmerman M, Spitzer RL (1989). Melancholia: from DSM-III to DSM-III-R. Am J Psychiatry.

[9] Parker G, Haxdzi-Pavlovic D (1996). Melancholia: A Disorder of Movement and Mood.

[10] Parker G, Fink M, Shorter E (2010). Issues for DSM-5: whither melancholia? The case for its classification as a distinct mood disorder. Am J Psychiatry.

[11] Kendler KS (1997). The diagnostic validity of melancholic major depression in a population-based sample of female twins. Arch Gen Psychiatry.

[12] Joyce PR, Mulder RT, Luty SE (2002). Melancholia: definitions, risk factors, personality, neuroendocrine markers and differential antidepressant response. Aust N Z J Psychiatry.

[13] Melartin T, Leskela U, Rytsala H (2004). Co-morbidity and stability of melancholic features in DSM-IV major depressive disorder. Psychol Med.

[14] Klein DF (1974). Endogenomorphic depression. A conceptual and terminological revision. Arch Gen Psychiatry.

[15] Kessler RC (1997). The effects of stressful life events on depression. Ann Rev Psychol.

[16] Kendler KS, Kuhn JW, Prescott CA (2004). Childhood sexual abuse, stressful life events and risk for major depression in women. Psychol Med.

[17] American Psychiatric Association (1987). Diagnostic and Statistical Manual of Mental Disorders.

[18] Kendler KS, Gatz M, Gardner CO (2006). Personality and major depression: a Swedish longitudinal, population-based twin study. Arch Gen Psychiatry.

[19] Martin J, Anderson J, Romans S (1993). Asking about child sexual abuse: methodological implications of a two stage survey. Child Abuse Negl.

[20] Fink M, Bolwig TG, Parker G (2007). Melancholia: restoration in psychiatric classification recommended. Acta Psychiatr Scand.

[21] Parker G (2000). Classifying depression: should paradigms lost be regained?. Am J Psychiatry.

[22] Rush AJ, Weissenburger JE (1994). Melancholic symptom features and DSM-IV. Am J Psychiatry.

[23] Coryell W (2007). The facets of melancholia. Acta Psychiatr Scand Suppl.

